# Applications of Atomic Force Microscopy in HIV-1 Research

**DOI:** 10.3390/v14030648

**Published:** 2022-03-21

**Authors:** Itay Rousso, Akshay Deshpande

**Affiliations:** Department of Physiology and Cell Biology, Ben-Gurion University of the Negev, Beer Sheva 8443944, Israel; akshaygh@post.bgu.ac.il

**Keywords:** atomic force microscopy, HIV-1, mechanical properties, imaging

## Abstract

Obtaining an understanding of the mechanism underlying the interrelations between the structure and function of HIV-1 is of pivotal importance. In previous decades, this mechanism was addressed extensively in a variety of studies using conventional approaches. More recently, atomic force microscopy, which is a relatively new technique with unique capabilities, has been utilized to study HIV-1 biology. Atomic force microscopy can generate high-resolution images at the nanometer-scale and analyze the mechanical properties of individual HIV-1 virions, virus components (e.g., capsids), and infected live cells under near-physiological environments. This review describes the working principles and various imaging and analysis modes of atomic force microscopy, and elaborates on its distinctive contributions to HIV-1 research in areas such as mechanobiology and the physics of infection.

## 1. Introduction

Structural virology has become a field of increasing interest in recent years with the development of tools and techniques that enable researchers to study viruses as solid-state objects from a physical perspective. Investigating viruses and their infection mechanisms from this viewpoint opens an important avenue of research to study the mechanical properties of viruses, such as stiffness, elasticity, plasticity, and material fatigue. The use of traditional techniques, such as conventional electron microscopy, cryo-electron microscopy, X-ray crystallography, nuclear magnetic resonance spectroscopy, fluorescence, and circular dichroism have enabled researchers to determine the structure of viruses and other nano-molecules over the years. However, recent advances in physical and mechanical mapping techniques enable new light to be shed on virus structure and replication mechanisms. Extensive studies focused on the mechanical properties of enveloped and non-enveloped viruses have been summarized in several reviews. In the current review, we provide an overview of the physics and mechanics underlying HIV-1 infection.

## 2. Atomic Force Microscopy and Mechanical Measurements

**Figure 1:**
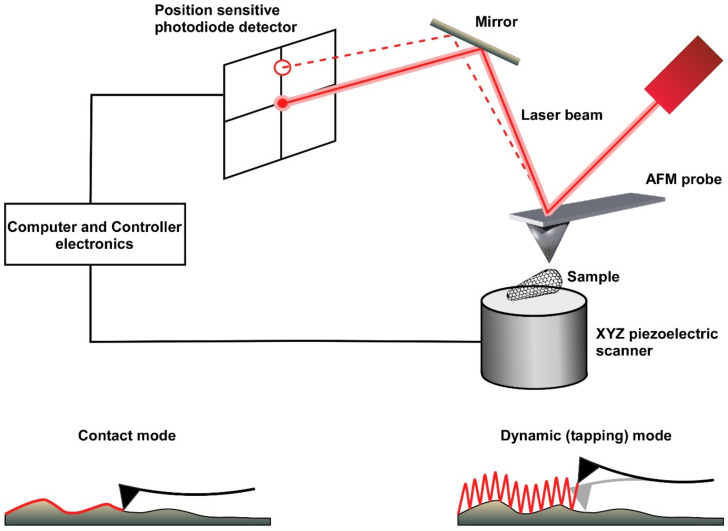
A schematic drawing of the main components of an atomic force microscope (AFM). The AFM is composed of an XYZ piezoelectric scanner, a laser source, a position-sensitive photodiode detector (PSD), and a controller. The AFM probe is a sharp tip that is attached to the end of a cantilever. A laser beam is reflected onto the PSD to detect small deflections of the cantilever as it scans the sample topography at very high force sensitivity. The two principal imaging modes of AFM, contact and dynamic modes, are schematically displayed. Contact mode is probably the simplest AFM imaging mode. In this mode, the probe is in continuous contact with the sample while scanning the surface at a constant force. In dynamic mode, the cantilever oscillates at or near the resonance frequency of the cantilever. As a result, the probe interacts with the sample as the probe “taps” along the surface, reducing the interactions between the sample and the probe. This mode is particularly powerful for imaging soft and gentle biological samples.

Advances in scanning probe microscopy were initially obtained from the development of scanning tunnelling microscopy by Binnig and Rohrer in the early 1980s [1]. To overcome the limitation that this technique could image only conducting or semi-conducting samples, an analogous technique of scanning probe microscopy, known today as atomic force microscopy, was developed in 1986 [2]. This technique utilizes atomic forces between the sample and the tip of the scanning probe. A sharp tip “feels” the surface of the sample, and by scanning the sample, a high-resolution topographical image is generated [3]. To maintain a constant force or a constant distance between the scanning probe and the sample, a laser beam is reflected from the probe cantilever onto a position-sensitive photodiode detector. A feedback loop mechanism between the detector and the z-piezo keeps the height of the probe above the sample constant during the entire image acquisition (Figure 1).

An atomic force microscope (AFM) can be used to construct topographic images (using its contact, dynamic, or quantitative modes) or to analyze surface forces per se (using its force mode), with both the quantitative and force modes relying on the production of force-versus-distance curves. In the contact imaging mode, the AFM probe scans the surface in a zig-zag pattern, thereby generating relatively high lateral (shear) forces on the sample. These forces may eventually damage either the sample or the AFM tip [4], thus, this mode of operation is not usually suitable for imaging biological samples, which are typically soft and more sensitive. In the dynamic imaging mode (also known as intermittent contact or tapping mode), a piezo crystal drives the cantilever to oscillate near or at its resonance frequency. As a result, imaging is obtained from a series of intermittent taps on the sample surface that drastically reduce the tip–sample contact time, thereby minimizing damage to both the sample and the tip [5]. More recently, a new quantitative imaging (QI) mode has been developed for gentle non-destructive imaging of soft samples. The QI imaging mode involves producing force-versus-distance curves (also called force curves) by controlling the vertical application of force at every pixel of the image while minimizing the lateral shear force generated from the horizontal movement of the tip. Force-curve-based imaging not only generates topographical images with high resolution but also permits the extraction of physical sample parameters, such as local stiffness, elasticity and adhesion [6]. In addition to its imaging functions, the AFM can be operated in force mode to assess the mechanical properties of the sample. In this mode, the probe is positioned at a fixed location and then repetitively pushed against and retracted from the sample. During each cycle, the elastic response of the probe cantilever is recorded in the form of a force–distance curve. From these force–distance curves, sample properties, such as point stiffness, elasticity, plasticity, adhesion, and mechanical strength, can be derived [7].

One of the main advantages of atomic force microscopy as a high-resolution imaging technique is its ability to analyze non-fixed samples in fluid environments; thus, it is capable of measuring biological samples under near-physiological conditions. However, samples must be adhered or immobilized to a supporting surface. Thus, much care must be taken when immobilizing a soft and delicate biological sample so that it is attached sufficiently strongly to the substrate to allow successful scanning without impairing its native morphology and structure. Depending on the sample, a wide range of substrates (e.g., mica, graphite or glass coated with poly-lysine or with hydrophobic hexamethyldisilazane (HMDS)) can be used to adhere the biological samples to the substrate.

## 3. Atomic Force Microscopy in Virology

Atomic force microscopy is finding increasing applications in the study of the mechanical and physical properties of biological samples (reviewed in [7]). The ability of the AFM to image untreated samples in fluid under near-physiological conditions and at high spatial resolutions makes it a powerful tool in structural and molecular biology, while its ability to visualize structural changes at the single particle level has made it a valuable tool for exploring the nano-mechanics governing biological processes (reviewed in [8]). External conditions can easily be manipulated, thus enabling researchers to follow highly regulated biological processes involving a myriad of complex biomolecules, such as protein aggregates, virus capsid assembly, biopolymers, cytoskeletal infrastructure, cells, and tissues, using atomic force microscopy in combination with conventional microscopy techniques and advanced optical methods (reviewed in [9,10]).

In virology, atomic force microscopy has been widely used to image single virus structure as well as individual virus components. In parallel, it has been used to study the mechanical properties of virus particles as well as interaction forces between viruses and their physical environment. In bacteriophages, atomic force microscopy studies have revealed the unique properties of the viral shell of bacteriophages (called the capsid), in which nucleic acid is tightly packed prior to being injected under extremely high-pressure into a bacterial cell during infection. Bacteriophage capsids are relatively stiff to protect the viral genome but also brittle, which makes them susceptible to failure. Interestingly, bacteriophages overcome the brittleness problem during capsid maturation by initially forming thin-walled capsids while maintaining intermolecular integrity [11]. Similarly, eukaryotic viruses form a mechanically rigid and pressurized structure that may withstand large deformation without undergoing any significant structural failure [12]. By contrast, some eukaryotic viruses and virus capsids demonstrate an anisotropic distribution of stiffness and elasticity along the capsid structure, thus permitting them to withstand shear forces during rapid movement through viscous physiological fluids or while passing through narrow openings such as nuclear pores [7,13,14,15,16].

The fine balance of mechanical properties exhibited by viral particles during the course of infection makes the field of physical virology an interesting area to explore. Since viruses and virus capsids are biologically soft in nature, it becomes imperative to understand the mechanics governing the physical properties of these small entities, especially during infection, which involves overcoming several harsh physical and chemical barriers. Extensive studies focused on the mechanical properties of enveloped and non-enveloped viruses have been summarized in several reviews [4,6,7,8,10,17,18]. In this review, we provide an overview of the physics and mechanics underlying HIV-1 infection.

## 4. Human Immunodeficiency Virus Type 1

**Figure 2:**
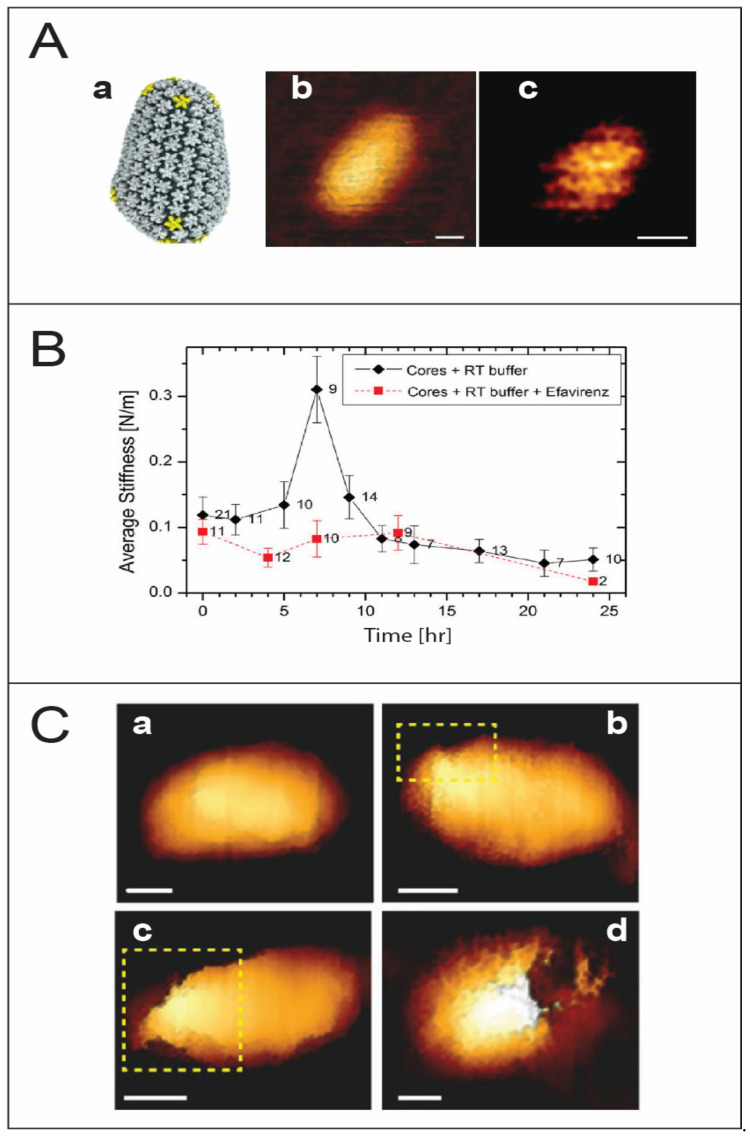
HIV-1 uncoating. (**A**) High resolution topographic imaging of wild type conical capsid protein assemblies using atomic force microscopy. (**a**) The molecular architecture of the core derived from electron tomography (taken from [21]). (**b**) An image of the surface of a conical capsid showing a honeycomb lattice on its surface. (**c**) Scanning the top region of the conical capsid reveals the capsid lattice structure. Scale bars are 20 nm (taken from [22]). (**B**) Changes in the average stiffness values of HIV-1 cores during reverse transcription in the absence or presence of the reverse transcription inhibitor efavirenz (taken from [23]). (**C**) The morphology of IP6 treated HIV-1 core as a function of reverse transcription time. (**a**) A representative cone-shaped core prior to reverse transcription. (**b**–**d**) Deformed and damaged cores visualized after 5 h of reverse transcription. Openings in the capsid in the topographic AFM images are identified by enclosure within a dashed yellow rectangle. Scale bars, 50 nm (taken from [24]).

HIV-1 is an enveloped lentivirus studded with viral gp120/gp41 trimers. The lipid bilayer acquired during budding has a relatively low copy number of envelope protein moieties [19,20]. Underneath the viral membrane, the virus has a shell composed of the matrix protein. The characteristic conically shaped viral core (Figure 2A), which is made of a lattice of mostly capsid hexamers and 12 pentamers, contains two copies of genomic RNA as well as reverse transcriptase and integrase enzymes.

HIV-1 infection begins with binding of the virus to target cells via interaction between the viral gp120 envelope protein and the cellular CD4 receptor and co-receptor CCR5 or CXCR4. This binding results in fusion between the viral and cell membranes, which permits entry of the viral core into the target cell. The viral core must travel through the viscous and densely packed cytoplasm of the target cell to the cell nucleus and dock at the narrow nuclear pore [25,26,27,28]. To successfully infect the target cells, the viral genome, which is in the form of ssRNA, must be reverse transcribed into dsDNA and integrated with the host chromosomal DNA. Release of genetic material from the core requires disassembly of the core capsid shell and is referred to as uncoating. Next, viral proteins are transcribed from the integrated viral genome and then transported to the vicinity of the cell membrane, where they assemble into a newly formed spherical viral particle that buds from the host cell. After budding from the cell, HIV-1 undergoes a maturation process that is required for infectivity. During maturation the viral Gag protein is cleaved into the structural proteins (matrix, capsid, nucleocapsid), and the virus acquires its mature-state morphology.

The various stages of the HIV-1 life cycle have been extensively studied in detail using multiple biochemical and fluorescence techniques. From a biophysics perspective, the virus particles must overcome mechanical forces during both entry and exit from target cells. There are multiple points in the complete process at which they are vulnerable to loss of structural integrity and failure. In the proceeding sections, we will describe studies that utilized atomic force microscopy to provided new insights into the fundamental biophysical mechanism at the following mechanical checkpoints in the HIV-1 life cycle: viral entry, maturation, uncoating, and budding.

## 5. HIV-1 Mechanical Properties, Maturation and Entry

Unlike other viral genomes [13,29,30,31,32], the HIV-1 genome is not maintained under pressurized conditions. Thus, the rationale for studying the mechanical properties of HIV-1 particles is not immediately apparent. However, the virion must satisfy several potentially conflicting demands during its lifetime: durability during budding and in the outside environment to protect the genomic cargo and sufficient malleability to enable membrane fusion during entry into the target cell. It is therefore reasonable to assume that the virus must adopt a different set of physical properties at different stages of its life cycle.

The mature and immature states of the virus have different morphologies, with the thickness of the protein inner shell significantly greater in the immature state [33]. In an initial study on gammaretroviral murine leukemia virus (MLV) particles, the immature state was twofold stiffer than the mature state [34]. Moreover, finite element simulation showed that the immature virus shell can absorb local external stresses, thus potentially providing better protection to the internal genome than the mature shell. A more dramatic stiffness switch is observed during HIV-1 maturation, with the immature particle being 14-fold stiffer compared with the mature virus particles [35]. Previous studies had shown that immature virions with high stiffness have low entry activity and infectivity, in contrast to mature virions [36]. Surprisingly, the absence of the complete envelope protein (Env) drastically reduces the stiffness of immature virus particles. Specifically, deletion of the long cytoplasmic (CT) domain of Env results in the same drastic drop in the stiffness of the immature virion, indicating that the presence of the Env CT domain, rather than the thickness of the protein shell, contributes to particle stiffness [35]. This is an unexpected result, given the presence of limited copies of Env on the surface of the virion [19,20,37,38,39,40]. Intriguingly, immature virions without the Env CT domain demonstrate higher infectivity, thus suggesting an inverse correlation between viral stiffness and entry activity. Separating the CT domain and Env ectodomain revealed that the former regulates stiffness whereas the latter regulates entry, and that particle stiffness and virus entry are inversely correlated. Since HIV-1 gp41 undergoes significant conformational change to achieve membrane fusion, it is probable that stiffer virus particles present a higher energy barrier to membrane deformation during fusion, thus concurrently failing to enter [41]. Therefore, it is logical to state that mechanical properties are as important as biological interactions in governing the infection process. Unravelling the stiffening mechanism of the Env CT-domain remains an important goal for future research.

## 6. The Mechanics of Virus Uncoating

Over the years, three models have been proposed for virus uncoating: (1) immediately upon entry into the cytoplasm [42,43,44]; (2) in the vicinity of the nuclear pore; and (3) based on recent evidence, within the nucleus, which requires entry of a nearly intact core through the nuclear pore [45,46,47,48,49,50,51,52,53]. The core must remain structurally intact in order to protect its cargo from cellular factors, yet be malleable enough to disassemble at the required time and location to release the viral genome. Thus, the mechanical properties of the viral core are pivotal for infectivity.

Stiffness measurements reveal that in vitro HIV-1 wildtype capsid assemblies have lower stiffness values compared with cores isolated from purified wildtype virions [22]. This difference in stiffness may be indicative of the adaptability of virus mechanical properties in response to their contents. Since the supercoiled structure of ssRNA is not sufficient to completely occupy the entirety of the core volume [54], interactions between the genome and the inner walls of the core are probable, as has previously been described in the case of DNA viruses, which have a highly rigid hydrated dsDNA structure, leading to higher stiffness values [7]. Capsid-stabilizing mutations significantly stiffen the capsid structure, which is inversely correlated with the decreased infectivity of these mutations. Similarly, addition of the host cell factor cyclophilin-A increases HIV-1 capsid stiffness and decreases infectivity [55,56]. The E45A/R132T double mutant, however, breaks the above inverse correlation. This double mutant has a stiffness similar to that of the non-infectious E45A capsid [22], but was shown to partially restore viral infectivity [57]. This discrepancy indicates that the role of capsid mechanical properties in viral infectivity is far more complicated than the above naïve picture according to which viral infectivity and core stiffness are directly correlated.

The nature of the mechanism governing the location and timing of uncoating remains a pivotal question. Several studies suggest a correlation between capsid stability, uncoating, and viral infectivity [57,58,59,60,61]. Furthermore, capsid uncoating and reverse transcription are thought to be coupled. Based on the above, an intriguing proposal was put forward according to which reverse transcription of the relatively flexible ssRNA to a more rigid dsDNA creates enough pressure from within to trigger core uncoating. In pioneering sets of experiments, multiple and individual HIV-1 virus cores were monitored over the time course of reverse transcription. This time-lapse monitoring of virus cores revealed a progressive rise in stiffness to reach a maximum value almost 3-fold higher than the initial stiffness, followed by a sharp drop in stiffness and subsequent disassembly within 20 to 24 h of reverse transcription [23] (Figure 2B). The rise in capsid stiffness can be attributed to an increase in the internal pressure, which eventually triggers disassembly of the core. If the mechanical stability of the core is increased by a point mutation in the capsid protein, the generated internal pressure is not sufficient to break the core. Alternatively, if the induced internal pressure is reduced, for example in an RT-defective mutant, core disassembly is blocked. Cumulatively, the above results point to a delicate biophysical balance between the mechanical properties of the metastable virus core and reverse transcription-induced internal pressure. This interplay probably occurs during the transit of the core from the cell periphery to the nucleus, while interacting with a wide range of cellular factors and yet preventing detection by cellular defense mechanisms.

Interestingly, AFM analysis reveals that disassembly is observed to begin always near the narrow end of the core, where the local density of capsid pentamers is highest [21,62,63] (Figure 2C). However, recent studies using EM show that the virus capsid disassembles by losing patches of the capsid lattice [64]. PF74, a small molecule that specifically interacts with hexamers in the capsid lattice to stabilize them while destabilizing the pentamers, causes a dose-dependent increase in the stiffness of wildtype HIV-1 cores to levels similar to that of the hyperstable capsid mutant E45A. Nonetheless, PF74-treated cores show characteristic partial reverse transcription-induced disassembly at the narrow end of the core [65]. Further supporting evidence was provided by a transmission electron microscopy (TEM) and cryogenic electron microscopy (Cryo-EM) imaging study showing that the initial stages of uncoating involve the loss of relatively small patches to accommodate the growing length of the stiffer viral DNA [64]. Lentiviruses are characterized by conical-shaped cores and are more efficient at infecting nondividing cells, and their infectivity depends on capsid stability. It is therefore tempting to speculate that the cone-shaped cores contribute to lentivirus infection by introducing inherently structurally weak regions.

To better understand the uncoating mechanism in vivo, it becomes imperative to understand the role of cellular factors that interact with the virus core during uncoating. A major player in this regard is IP6 (inositol-hexakisphosphate), which has been shown to stabilize virus cores by interacting with capsid hexamers and pentamers [66]. Approaches using molecular dynamics (MD) simulations, AFM, TEM, confocal microscopy, and virus infectivity assays have been employed to better understand the role of IP6.

Mechanical studies on IP6-treated virus cores showed that their initial stiffness is elevated to a value similar to the E45A hyperstable mutant. However, cores undergo complete disassembly after a relatively short time (~7 h) of reverse transcription without displaying the characteristic stiffness peak [24]. Time-lapse monitoring of individual IP6-treated wildtype virus cores granted a better temporal resolution of the rapid process and showed the presence of not one but at least three individual stiffness spikes. A parallel study of an RT-defective mutant revealed the presence of just one spike in stiffness, thus providing novel preliminary links between stiffness spikes and reverse transcription stages. Using efavirenz, an RT-inhibitor, it was shown that all three spikes are necessary for successful core disassembly [67]. The rapid biomechanical changes fit well with the existing hypothesis, accounting for the struggles of the virus core to enter the nucleus through the narrow nuclear pore. Based on the above results, an accumulative damage model was put forward. According to this proposed model, mechanical changes lead to the formation of small ruptures on the core surface. The accumulation of these ruptures on the virus core surface eventually results in loss of the structural integrity of the core and ultimately triggers capsid disassembly [68]. Our above proposed model for core disassembly was recently supported by a cryo-tomography imaging of virus cores [69]. It was shown that HIV-1 cores undergoing active reverse transcription have higher lattice strain, which increases until an ultimate mechanical failure of the virus core. The crack propagation occurs along the high strain regions on the capsid lattice, thus showing how the mechanical properties are critically involved in core disassembly. The acceleration of reverse transcription kinetics in the presence of IP6, on the other hand, shows how HIV-1 has evolved particularly to utilize cellular IP6 to enhance the importation of nucleotides while it is in the cytoplasm, where their concentration is relatively low compared to in the nucleus.

The above in vitro experiments provide insight into the material properties of virus cores, but it is important to understand how these attributes are relevant and what role they play during the actual infection process in vivo. The increase in stiffness can be attributed to the presence of rigid nucleic acid within the capsid, but could also be due to the generation of free pyrophosphates (PPi) that phosphorylate the inner wall of multiple virus capsids, causing minor structural changes [70,71,72,73]. Similarly, infectivity studies have shown an increase in the stiffness or stability of the virus core due to point mutations; however, this increase does not necessarily correlate with reduced infectivity in all cases [22,57]. This challenges the dictum that high core stiffness reduces infectivity and points to a more intricate relation between the biophysical properties of the virus core and the mechanics of uncoating. Furthermore, multiple questions remain unanswered in this regard. Are these small reverse transcription-induced fractures reversible, thus preventing premature disassembly? What actually triggers the final stage of uncoating in the HIV-1 virus core? It would be interesting to approach these questions from a biophysics perspective by exploring the innate mechanical properties of the HIV-1 core.

## 7. HIV-1 Budding

In HIV-1, budding occurs near the plasma membrane where the virus assembles all the necessary viral components and buds out while acquiring a part of the plasma membrane. The primary driving force for budding is the Gag protein, which regulates all the activities for assembly and egress of the virus. Although electron microscopy can generate images of viral budding at excellent resolutions, it is not possible to obtain real-time kinetics data using this methodology. Since HIV-1 budding occurs near the cell membrane [74,75], it creates a perfect scenario for atomic force microscopy. Probing the cell membrane in real time can provide detailed information on the kinetics of individual budding events.

**Figure 3:**
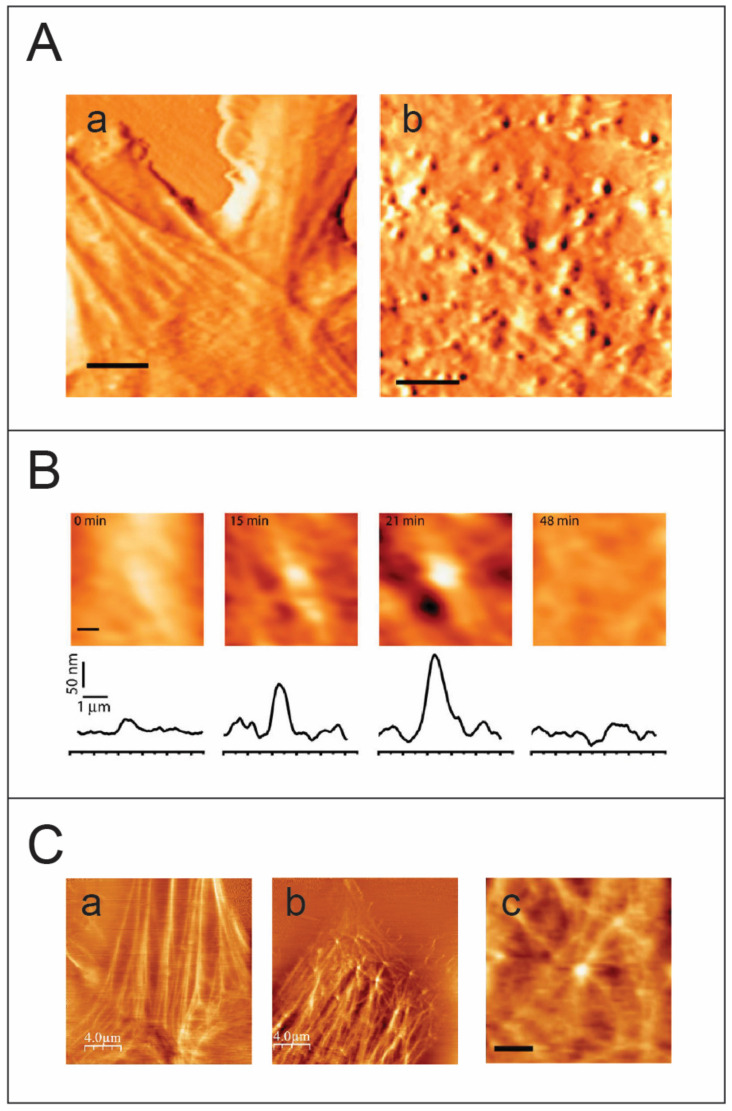
Imaging retrovirus budding from live cells using atomic force microscopy. (**A**) AFM images, acquired in tapping mode, of an (**a**) uninfected and (**b**) HIV-1 infected NIH/3T3 live cell. Virus particles at different stages of budding appear as protrusions of different heights on the cell membrane. Scale bar is 3 μm. (**B**) Visualizing a complete budding progress from initial assembly to release of a single virus particle. Scale bar is 0.5 μm. (**C**) Imaging the cytoskeleton of live cells using the AFM in the torsional mode. (**a**) an AFM image of an uninfected live cell. The cytoskeleton fibers are clearly detected beneath the cell membrane. (**b**) An AFM torsion image of an HIV-1 infected cell reveals the fine actin architecture beneath the cell membrane (an enlargement is shown in (**c**), scale bar is 1.5 μm). The virus particles colocalize to form actin star-like shapes. Panels A and B are taken from [76], while C is taken from [77].

In an initial study, individual MLV budding events were monitored using an AFM [76] (Figure 3). The accumulation of Gag protein under the cell membrane and its subsequent multimerization lead to the development of curvature in the cell membrane. Formation of a complete spherical Gag particle can be detected as a protrusion on the cell surface (Figure 3A), and the cross-sectional height determined by the AFM images over time provides details about the trajectory for budding. Single particle analysis provides insights into different budding patterns and characterizing these patterns contributes to assessing budding kinetics (Figure 3B). The complete budding trajectory can be divided into two parts: the growth of the bud and the excision of the bud to release the virion. Interestingly, two kinetic pathways were observed: a fast kinetics pathway characterized by the growth of the particle to maximal height followed by rapid release, and a slow pathway that includes an additional arrested phase between the growth and the release of the virus particle. Speculation that the arrest phase represents entrapment of the virion at the plasma membrane was resolved when the force applied during AFM image acquisition did not trigger release of the virus particle from the plasma membrane, thus indicating that the arrested phase is an intrinsic property of slow kinetics budding events [76]. Since growth phase kinetics are similar in the fast and slow pathways, it is possible that the arrest phase correlates with recruitment of the cellular machinery to trigger the final nicking of the bud. A fascinating question therefore arises as to whether poor recruitment or low local concentration of cellular machinery proteins is responsible for stalling in the arrest phase.

Several studies on equine infectious anemia virus (EIAV), vaccinia virus, MLV, and HIV, show the participation of actin in remodeling the cytoskeleton during viral budding [78,79,80,81,82,83,84]. Multiple approaches using fluorescence, AFM, and TEM have revealed large scale changes in cellular infrastructure with concurrent impacts on cellular morphology [85,86,87,88]. To better resolve the cytoskeleton of the cell, the AFM is operated in the torsional force mode. In this mode, the AFM probe scans perpendicularly to the long axis of the cantilever. As the tip bumps into a cytoskeletal fiber, which is stiffer than the cell membrane, the cantilever twists slightly, giving rise to an increase in the torsional signal. Operating the AFM in the torsional mode, HIV-1 and MLV budding events showed highly dynamic actin-filaments emerging from viral sites, thus providing direct visual links to the involvement of actin in retroviral budding (Figure 3C). Although the HIV-1 viral load produced is less in NIH 3T3 cells, the HIV-1 particles released are completely infectious and budding occurs relatively faster compared with that of MLV [77]. Parallel data from theoretical studies describing membrane proteins suggests a dynamic instability transition effect due to multimerization of proteins under the fluid cell membrane leading to formation of convex structures that eventually bud outwards [89,90]. When considered together with studies on Gag-actin interaction during budding [91], the observations cumulatively point to the role of actin as a cellular trigger for retroviral budding. Presently, the involvement of actin polymerization in HIV-1 budding is subject to debate, with several studies supporting this idea [78,92] while others suggest otherwise [92,93,94,95,96]. The results from atomic force microscopy and fluorescence microscopy studies lead to speculation regarding an interesting model in which viral Gag-proteins need to multimerize to a critical density and form a convex spherical shape to induce membrane instability and eventually trigger the formation of a bud. In addition, based on data acquired from AFM imaging combined with in vitro and theoretical studies, it is suggested that viruses utilize the cellular mechanical force generated by cytoskeletal assembly to overcome the energetic barrier associated with budding [77].

Analysis of the relatively fast kinetics of HIV-1 budding using standard atomic force microscopy revealed that its poor temporal resolution constitutes a weakness of this method. With the recent development of fast scanning AFMs capable of imaging soft biological samples with a temporal resolution of a few seconds, detailed characterization of HIV-1 budding has become possible.

## 8. Summary and Perspectives

After more than three decades of development in the area of near-field microscopy, atomic force microscopy has emerged as a powerful and significant tool to image and study the mechanical properties of a wide range of biological systems. By combining force microscopy and spectroscopy, atomic force microscopy opens a doorway to characterizing important biological processes from the novel perspective of biophysics and mechanobiology. Atomic force microscopy grants key insights into the mechanical properties governing fundamental biological processes and how their physical characteristics bestow different functional advantages on small molecular entities, such as protein clusters or viruses, at different time points in their development.

As elucidated by this review, atomic force microscopy provides uniquely relevant images and physical information concerning the mechanobiological properties of biological systems. Complementing traditional techniques, such as electron microscopy, X-ray crystallography, and mass-spectroscopy, atomic force microscopy greatly furthers our understanding of critical biological processes by examining their underlying physics.
